# Deciphering the Stepwise Binding Mode of HRG1β to HER3 by Surface Plasmon Resonance and Interaction Map

**DOI:** 10.1371/journal.pone.0116870

**Published:** 2015-02-06

**Authors:** Carmen Peess, Leopold von Proff, Sabine Goller, Karl Andersson, Michael Gerg, Magnus Malmqvist, Birgit Bossenmaier, Michael Schräml

**Affiliations:** 1 Roche Diagnostics GmbH, Penzberg, Germany; 2 Roche Innovation Center, Penzberg, Germany; 3 Ridgeview Diagnostics AB, Uppsala, Sweden; Irvine, UNITED STATES

## Abstract

For the development of efficient anti-cancer therapeutics against the HER receptor family it is indispensable to understand the mechanistic model of the HER receptor activation upon ligand binding. Due to its high complexity the binding mode of Heregulin 1 beta (HRG1β) with its receptor HER3 is so far not understood. Analysis of the interaction of HRG1β with surface immobilized HER3 extracellular domain by time-resolved Surface Plasmon Resonance (SPR) was so far not interpretable using any regular analysis method as the interaction was highly complex. Here, we show that Interaction Map (IM) made it possible to shed light on this interaction. IM allowed deciphering the rate limiting kinetic contributions from complex SPR sensorgrams and thereby enabling the extraction of discrete kinetic rate components from the apparently heterogeneous interactions. We could resolve details from the complex avidity-driven binding mode of HRG1β with HER3 by using a combination of SPR and IM data. Our findings contribute to the general understanding that a major conformational change of HER3 during its activation is induced by a complex sequential HRG1β docking mode.

## Introduction

The HER receptor family is an important target for cancer therapeutics (reviewed by [1]). In order to develop even more potent treatment options than currently available, it is indispensable to understand the mechanism of the HER receptor activation. In contrast to HER2, where no ligand is known and which is present on the cell surface in a constitutively open and active conformation [2], the other members of the HER family depend on ligand-induced activation (reviewed by [3]). It is meanwhile accepted that the activation modes of the HER family receptors by diverse growth and differentiation factors are characterized by tremendously complex molecular rearrangements [4]. In its closed conformation the domains II and IV of the HER3 receptor make contacts via several amino acids and thereby form a so called intramolecular ‘tether’ [5]. In this tethered conformation the HRG1β interaction sites, comprised of domains I and III, are too distant from each other to allow simultaneous HRG1β binding [5]. This intramolecular tether has to be dissipated and a major conformational rearrangement has to take place, in order to allow the ligand to simultaneously contact domains I and III, resulting in the stabilization of the open receptor conformation [4, 6, 7]. Whether this conformational rearrangement is solely induced by ligand binding [7] or if the ligand docks into an already adopted and spontaneously pre-formed receptor conformation [8], is still under discussion. Ligand binding finally results in receptor homo- or heterodimerization with other HER family receptor members [9–11], which induces activation of downstream signaling pathways [12].

The binding modes of the HER ligands to their receptors remain to be elucidated by more powerful methods than currently available. The majorities of functional assays only shed light on the end point equilibrium formation, but omit the series of important, dynamic molecular binding events, taking place on the way to the equilibrium. This results in a profound lack of knowledge about the intermolecular dynamics, which could harbor a tremendous source of information for the development of more efficient diagnostic reagents and effective pharmaceuticals. Real-time interaction measurements have high information density, especially in the association phase kinetics of the interacting molecules [13]. So far, there were no technical means available to take advantage of the apparently complex experimental data, through extracting the multiple kinetic rate constants embedded in one single set of experimental data. Interaction Map (IM) [14] is a novel analysis method suitable to break down the parallel binding events present in complex time-resolved interaction data from biophysical measurements [15] and cell-based assays [16, 17]. Here, we applied IM to gain more information from complex and heterogeneous real-time surface plasmon resonance (SPR) data to investigate the dynamics of the HER3/HRG1β interaction. We show that HRG1β first contacts HER3, in an initial docking event, at a single binding site. In a second step, HRG1β contacts with its second HER3 binding site. This synergistic binding results in a high affine, avidity-driven interaction between HRG1β and its two HER3 epitopes (domain I and III). These findings were made possible by applying the novel Interaction Map technology to extract relevant kinetic components from complex SPR real-time data. From a general perspective, our results show the enormous advantage of combining SPR interaction data with tools that reveal a deeper insight into complex kinetic components.

## Materials and Methods

### Interaction measurements using Surface plasmon resonance

Antibodies were named according to Baumgarten and Kürzinger [18]. For the collection of surface plasmon resonance data at 25°C, a Biacore T200 instrument (GE Healthcare) was used. Two assay setups on two different sensor chips were designed. The first assay setup was built on a series S senor chip CM5 (GE Healthcare). An antibody capture system was established on the sensor surface. 10 000 RU MAb<M-IgG>Rb (BR-1008-38, GE Healthcare) were immobilized on each flow cell, using ECD/NHS chemistry following the instructions of the manufacturer. The chip was saturated with 1 M ethanolamine. The system buffer was 10 mM HEPES pH 7.4, 150 mM NaCl, 1 mM EDTA, 0. 5% (v/v) Tween 20. The system buffer was supplemented with 1 mg/ml carboxymethyldextran (Fluka), to obtain sample buffer.

In assay A, B and C, flow cell 1 was always used as a reference, using MAb<TSH>M-1.20-IgG (MW 150 kDa; 10767778103, Roche Diagnostics GmbH, Mannheim, Germany). For the assays A, B and C 205 RU of the ligand antibody MAb<hHER3-ECD>M-208 (mAb208; MW 150 kDa; pRED Roche Diagnostics GmbH, Penzberg, Germany) was first captured by injecting it for one minute at a flow rate of 10 µl/min. The analytes in solution were injected at a flow rate of 60 µl/min. For assay setup A, human HER3 (MW 68 kDa; Dr. Birgit Bossenmaier, Roche Diagnostics GmbH, Penzberg, Germany), was injected in concentration series of 180 nM, 60 nM, 20 nM, 6.7 nM, 2.2 nM and 0 nM. For the assay B setup, identical concentrations of HER3 were used, but the analyte HER3 was pre-incubated for 2 hours at room temperature with a 1:6 HER3:HRG1β molar surplus of human heregulin β1 (HRG1β, MW 25 kDa; Dr. Birgit Bossenmaier, Roche Diagnostics GmbH, Penzberg, Germany) to pre-form HER3/HRG1β complexes. Subsequently in assay setup C HER3 was first bound to the antibody mAb208. Next, HRG1β was injected in a concentration series of 105 nM, 35 nM, 12 nM, 3.9 nM, 1.3 nM, and 0 nM at a flow rate of 60 µl/min. In all assay setups the association and dissociation phases of the respective analytes were monitored for 5 min and 10 min, respectively. The CM5 chip surface was fully regenerated with 1 injection of 10 mM Glycine pH 1.5 for 10 seconds and 2 injections of 10 mM Glycine pH 1.7 buffer for 30 seconds at 20 µl/min.

Assay setup D was built on a Biacore Biotin CAPture Kit (GE Healthcare). The coupling of the CAP sensor chip with streptavidin probes and the regeneration of the sensor surface were conducted according the manufacturer’s indications. The same sample buffer as already mentioned was used. For presenting the extracellular domain of HER3 via streptavidin on the sensor chip surface, the C-terminal Avi-Tag of HER3 was singly biotinylated (HER3-Avi-biotin, MW 70 kDa). It was a kind gift of pRED, Roche Diagnostics GmbH, Dr. Jürgen Schanzer). HER3-Avi-biotin was captured for one minute at a flow rate of 10 µl/min. No ligand was immobilized on flow cell 1, for reference purposes. Subsequently, HRG1β was injected at a flow rate of 60 µl/min. It was injected in a 1:3-series dilution at concentrations of 105, 35 nM, 12 nM, 3.9 nM, 1.3 nM, and 0 nM. The association and dissociation phases were monitored for 5 and 10 minutes, respectively.

Collected data were evaluated, using the Biacore T200 Evaluation Software 2.0 according to the manufacturer’s instructions. The Langmuir fit was adjusted to the full range of the association and dissociation intervals. In order to resolve complex interactions the SPR data were additionally evaluated using the TraceDrawer Software 1.6 (Ridgeview Instruments AB) and the obtained data was used to calculate an Interaction Map (Ridgeview Diagnostics AB) [14–16] by the Ridgeview Server Software. In brief, Interaction Map is capable of separating the signals from multiple parallel one:one interactions using a distribution based fitting approach. The measured curve is approximated with a sum of a range of primitive binding curves, each representing a one:one interaction [13] with a unique combination of association rate *k*_a_ and dissociation rate *k*_d_ (and consequently an equilibrium dissociation constant *K*_D_ = *k*_d_/*k*_a_). Each primitive curve has a weight factor and weights are fitted to make the sum of all primitive curves as similar to the measured curves as possible. The resulting weights are plotted in a surface plot as a function of *k*_a_ and *k*_d_. This plot is denoted an Interaction Map (IM). The currently used Interaction Map method uses 24 (*k*_a_) * 30 (*k*_d_) different nodes (with accompanying primitive curves) with kinetic parameter values evenly distributed in log-space.

Report points were used to additionally characterize the shape of the sensorgrams. CL_208_ is the capture level in response units of mAb208. CL_ECD3_ is the capture level in response units of HER3. BL_early_ is the binding signal in response units shortly before the end of the analyte injection. BL_late_ is the binding signal in response units 100 sec after the end of the injection. SL is the stability late signal in response units at the end of the dissociation phase. All report points were assigned to the highest analyte concentration. The Molar Ratio was calculated as the quotient of the molecular weights of the analytes or complexes in solution and the ligand on the sensor surface multiplied with the quotient BL_late_: CL_ECD3_. All assays were conducted three times and mean values of SPR and IM data as well as standard deviations were calculated of all replicates (n = 3). The third lowest analyte concentration was injected twice during each experiment, as an integrated injection control (n = 6).

To investigate the complex character of the four described interactions, a control experiment was conducted at 25°C, using the Biacore 4000 Evaluation software 1.0 (GE Healthcare). The system buffer was 10 mM HEPES pH 7.4, 150 mM NaCl, 1 mM EDTA, 0. 5% (v/v) Tween 20. The system buffer was supplemented with 1 mg/ml carboxymethyldextran (Fluka), to obtain sample buffer. The analytes were injected for 30 seconds and 300 seconds at a flow of 30 µl/min. The dissociation phase was monitored for 15 minutes. One flow cell with immobilized antibody MAb<CK-MM>M-33-IgG (MW 150 kDa; 11200941103, Roche Diagnostics GmbH, Mannheim; Germany) was used as a reference. The system was regenerated after each cycle, injecting 100 mM H3PO4 for 30 seconds. In assay A 180 nM HER3 were injected to 463 (±13) RU of immobilized mAb208 on a CM5 sensor chip. In assay B the pre-formed HER3/HRG1β (180 nM/1050 nM) complex was captured by 441 RU (±8) of immobilized mAb208 on a CM5 sensor chip. In assay C 105 nM HRG1β were injected after capturing 476 RU (±17) of HER3 by previously immobilized mAb208. In assay D 105 nM HRG1β were injected after capturing 561 RU (±25) of singly biotinylated HER3 on a CAP chip with streptavidin probes as described before. All assays were conducted three times.

## Results

**Figure 1:**
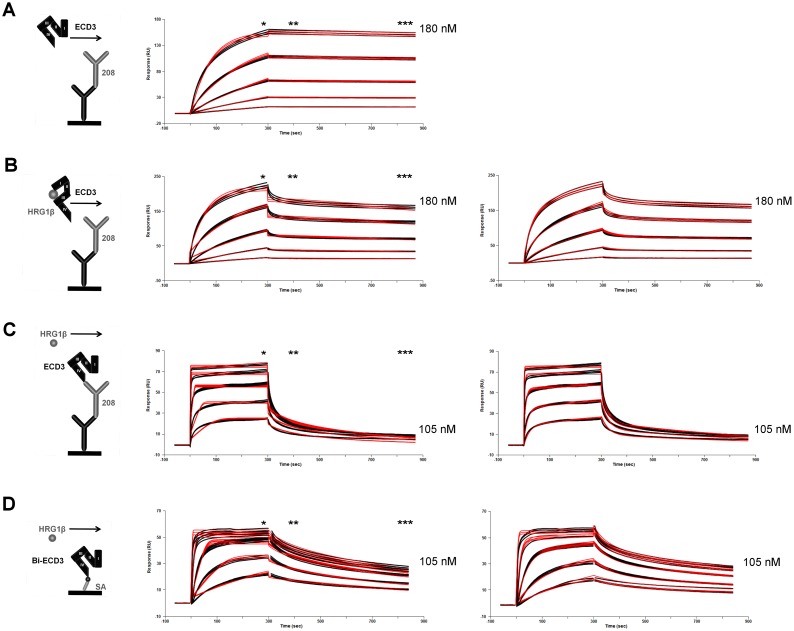
Deciphering the binding behavior of HRG1β to HER3, investigated by different SPR assay setups. To determine the interaction of HRG1β with HER3 in presence and absence of anti-HER3 antibody mAb208, four different SPR assay setups were designed. The descriptive symbols illustrate the corresponding assay setups. Arrows indicate injection of analytes (left column). Measured biomolecular interactions were evaluated using a regular Langmuir model (middle column) and where applicable using a two-state reaction model (right column) by Biacore Evaluation Software 2.0. The curve fittings are highlighted in red. The curve corresponding to the highest concentration is indicated in each sensorgram. Report points were used to additionally characterize the shape of the sensorgrams. They are indicated by asterisk: BL_early_ (*) is the binding signal shortly before the end of the analyte injection. BL_late_ (**) is the binding signal 100 seconds after the end of the injection. SL (***) is the stability late signal at the end of the dissociation phase. Three replicates of each concentration are shown in black in each sensorgram (n = 3). The third highest concentration of each assay was injected twice (n = 6). (A—C) Murine antibody mAb208 was captured by immobilized rabbit anti-mouse antibody on CM5 sensor chip surface. (A) Injection of HER3 (ECD3). (B) Injection of pre-incubated HER3/HRG1β. (C) Injection of HRG1β. (D) Biotinylated HER3-Avi (bi-ECD3) was captured on a streptavidin-coated CAP sensor chip. Subsequently, HRG1β was injected.

In order to set up an SPR assay format enabling the analysis of the HRG1ß/HER3 interaction we firstly analyzed the interaction of HER3 and the antibody mAb208 by SPR (Fig. 1A). MAb208 is a murine monoclonal antibody, specifically binding to domain IV of HER3. Therefore, mAb208 was captured with 256 RU on the sensor chip and HER3 was injected subsequently in various concentrations. The calculation of the Molar Ratio (MR 1.4) revealed functional 1:1 binding stoichiometry, meaning that one single HER3 bound to one mAb208 (table 1). The highest injection concentration (180 nM) of HER3 led to a signal amplitude of BL_early_ 156 RU with no significant signal shift after injection end (BL_late_ 150 RU). To calculate kinetic parameters, a 1:1 Langmuir data fit was applied on the SPR sensorgrams (Fig. 1A). Visual inspection of the overlay of the fitting curve and the measured sensorgram points to a fit with acceptable deviations in the association and dissociation phase, thus indicating a monophasic interaction. However, the fitting calculated a faster association of *k*_a_ 7.6E+04 M^-1^s^-1^ and a slower dissociation of *k*_d_ 4.9E-05 s^-1^ than the underlying experimental data indicated, leading to an apparent affinity calculation of *K*_D_ 0.7 nM. Despite a highest signal amplitude at R_max_ of only 158 RU the error of the fit (Chi^2^ 2.2 RU^2^; Chi ± 1.5 RU) was large in comparison to the short term noise level (approximately 0.2 RU peak to peak) meaning that the fit was not fully adequate. Despite some lacking fitting quality of the model overlay, such SPR data can be frequently found in typical biosensor publications [19]. The monophasic character of the mAb208/HER3 binding interaction was confirmed by a control complex interaction analysis (S1A Fig., S1 Text) [20].

**Table 1:** Kinetic interaction parameters calculated by Biacore.

**CL_208_**	**CL_ECD3_**	**BL_early_**	**BL_late_**	**SL**	**R_max_**	**MR**	***k*_a_**	***k*_d_**	***K*_D_**	**Chi^2^**	***K*_D_(2sr)**	**Chi^2^(2sr)**
**(RU)**	**(RU)**	**(RU)**	**(RU)**	**(RU)**	**(RU)**		**(M^-1^s^-1^)**	**(s^-1^)**	**(nM)**	**(RU^2^)**	**(nM)**	**(RU^2^)**
**Assay A**												
256 (±11)	-	156 (±5)	150 (±13)	150 (±5)	158 (±5)	1.36 (±0.02)	7.6E+04 (±6.3E+02)	4.9E-05 (±1.1E-06)	0.65 (±0.02)	2.2 (±0.2)	-	-
**Assay B**												
250 (±10)	-	222 (±6)	176 (±5)	161 (±4)	188 (±5)	1.21 (±0.02)	8.8E+04 (±1.8E+03)	2.8E-04 (±5.3E-06)	3.2 (±0.1)	9.0 (±0.6)	3.4 (±0.1)	1.9 (±0.2)
**Assay C**												
256 (±11)	211 (±3)	79 (±2)	22 (±2)	10 (±1)	43 (±2)	0.56 (±0.02)	1.2E+08 (±1.7E+07)	4.0E-02 (±6.1E-03)	0.33 (±0.01)	4.6 (±0.3)	1.6 (±0.1)	1.0 (±0.1)
**Assay D**												
-	179 (±9)	58 (±5)	42 (±4)	30 (±4)	56 (±4)	0.84 (±0.03)	1.2E+07 (±2.1E+06)	4.6E-03 (±4.4E-04)	0.38 (±0.04)	1.5 (±0.3)	1.0 (±0.1)	0.5 (±0.2)

Kinetic values of the four SPR assays calculated by Biacore (T200 Evaluation Software 2.0), using a 1:1 Langmuir fit. Additionally, the affinities (*K*_D_(2sr)) of SPR assays B, C and D were calculated using a two-state reaction (2sr) model. BL_early_, BL_late_ and SL are taken from the highest analyte concentration of each assay. RU: Response Units; CL_208_: Capture Level of mAb208; CL_ECD3_: Capture Level of HER3 and HER3-Avi-biotin; BL_early_: binding signal shortly before the end of the analyte injection; BL_late_: binding signal 100 sec after injection end. SL: is the stability late signal at the end of the dissociation phase. Listed are mean values of three replicates (±standard deviation).

**Figure 2:**
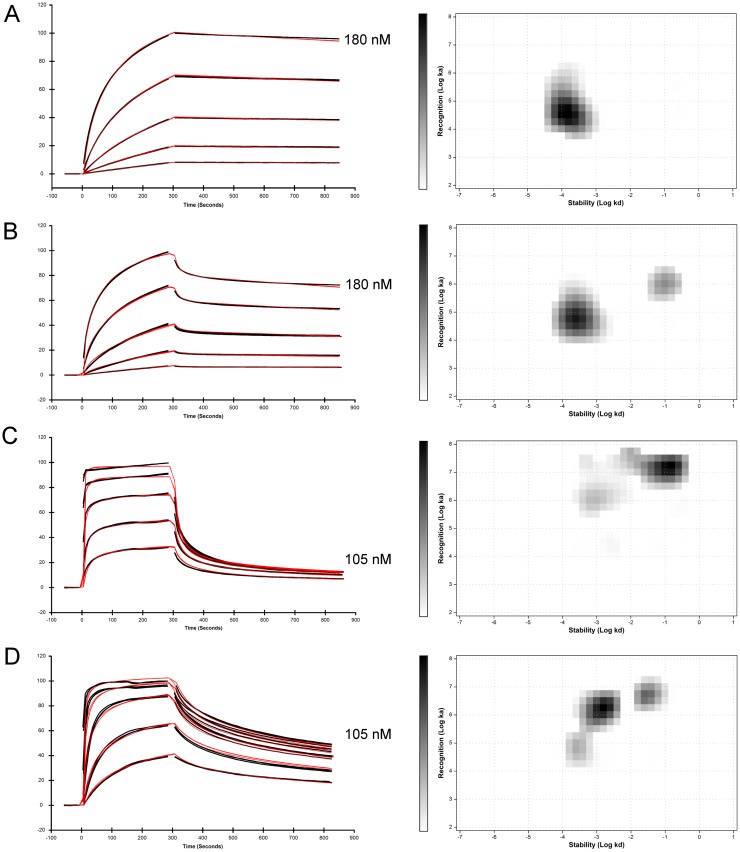
Deciphering the binding behavior of HRG1β to HER3, investigated by different SPR assay setups and analyzed by Interaction Map. To determine the interaction of HRG1β with HER3 in presence and absence of anti-HER3 antibody mAb208, the four SPR assays described in Fig. 1 were analyzed using Interaction Map (IM). The Multi-parameter fitting algorithm of IM was applied to the five concentrations used in the SPR assays (left column). The curve corresponding to the highest concentration is indicated in each sensorgram. The kinetic rate contributions are dissolved by IM (right column). Three replicates of each concentration are shown in black in each sensorgram. The third highest concentration of each assay was injected twice (n = 6). The applied IM fit was highlighted in red. (A—C) Murine antibody mAb208 was captured by immobilized rabbit anti-mouse antibody on CM5 sensor chip surface. (A) Injection of HER3 (ECD3). (B) Injection of pre-incubated HER3/HRG1β. (C) Injection of HRG1β. (D) Biotinylated HER3-Avi (bi-ECD3) was captured on a streptavidin-coated CAP sensor chip. Subsequently, HRG1β was injected.

To achieve more adequate data interpretation, we applied IM. Due to the multi-parameter fitting algorithm, the IM calculated fit (Fig. 2A) obviously aligned better with the underlying SPR data than the two-parameter Langmuir fit applied by the SPR control software (Fig. 1A). IM shows that the mAb208/HER3 interaction was strictly monophasic, with a single rate contribution of 80% (Fig. 2A) and thereby confirms the result of the complex interaction analysis (S1A Fig.). It calculates a *k*_a_ of 4.4E+04 M^‑1^s^-1^ and a *k*_d_ of 1.4E-04 s^-1^, which resulted in an affinity of *K*_D_ 3.1 nM (Fig. 2A, table 2). When compared with the kinetic results from the Langmuir model, IM showed a 4.8 times lower affinity. Whereas the Langmuir model calculates an apparent single set of kinetic constants, the IM affinity is ascribed directly to the center of the IM spot. Therefore, the values reported by IM will differ from a Langmuir model in a similar manner as comparing median and average on data from non-uniform distributions.

**Table 2:** Kinetic interaction parameters calculated by Interaction Map.

***k*_a_**	***k*_d_**	***K*_D_**	**Weight**
**(M^-1^s^-1^)**	**(s^-1^)**	**(nM)**	**(%)**
**Assay A**			
4.4E+04 (±1.7E+03)	1.4E-04 (±1.1E-05)	3.1 (±0.4)	79.8 (±1.2)
**Assay B**			
6.3E+04 (±8.4E+02)	3.0E-04 (±1.7E-05)	4.7 (±0.3)	64.6 (±0.9)
1.0E+06 (±4.5E+04)	1.1E-01 (±7.9E-03)	110 (±9.3)	17.6 (±0.4)
**Assay C**			
1.5E+07 (±2.9E+05)	8.9E-02 (±3.5E-03)	6.1 (±0.3)	62.8 (±1.4)
1.5E+06 (±2.5E+05)	1.2E-03 (±1.2E-04)	0.9 (±0.1)	15.9 (±1.3)
**Assay D**			
1.4E+06 (±5.2E+05)	1.4E-03 (±6.0E-04)	1.2 (±0.5)	54.3 (±5.9)
4.2E+06 (±9.0E+05)	2.5E-02 (±9.6E-03)	6.8 (±3.8)	26.9 (±6.4)
6.0E+04 (±1.9E+04)	2.7E-04 (±3.8E-05)	4.9 (±1.2)	12.3 (±2.1)

Kinetic values of the four SPR assays shown in table 1, calculated by Interaction Map (TraceDrawer Software 1.6, Ridgeview Instruments AB). Listed are mean values of three replicates (±standard deviation).

To investigate whether the presence of HRG1β affects the complex formation of mAb208/HER3, we performed SPR analyses, where pre-incubated HER3/HRG1β complex was injected as a ligand (Fig. 1B). 250 RU mAb208 were captured on the sensor chip followed by injection of the ligand. Visual inspection of the sensorgram identified a 46 RU signal drop from BL_early_ 222 RU to BL_late_ 176 RU and to SL 161 RU at the end of the dissociation phase. The ligand showed a 1:1 binding stoichiometry of MR 1.2 to mAb208. The Langmuir fitting algorithm calculated a *k*_a_ of 8.8E+04 M^-1^s^-1^, a *k*_d_ of 2.8E-04 s^-1^ and a *K*_D_ of 3.2 nM with a significant failure of Chi^2^ 9 RU^2^ (Fig. 1B). The evaluation software interpreted the signal drop as a bulk effect, so that the kinetic evaluation data within this time corridor was missing. Very probable, this produces misleading data. In order to justify a fit of higher complexity, the complex character of the mAb208 interaction with the pre-formed HER3/HRG1β was proven by a control experiment (S1B Fig.). The affinity calculated by the applied two-state reaction fit was *K*_D_(2sr) 3.4 nM. The Chi^2^(2sr) value of the two-state fit decreased to 1.9 RU^2^ in comparison to the Langmuir fit Chi^2^ (9.0 RU^2^) (table 1).

To decipher this complex kinetic, we applied IM. Again, the IM calculated fit (Fig. 2B) aligned obviously better with the underlying SPR data than the applied Langmuir fit (Fig. 1B). However, it was comparable with the quality of the SPR two-state fit. IM delivered new and more detailed information than SPR and was able to resolve two discrete kinetic components (Fig. 2B). The dominant circular spot contributed to 65% of the overall interaction and indicated a homogenous 1:1 affinity of *K*_D_ 4.7 nM composed from a *k*_a_ of 6.3E+04 M^-1^s^-1^ and a *k*_d_ of 3.0E-04 s^-1^, which was comparable to the affinity *K*_D_ of 3.1 nM (table 2) and *K*_D_(2sr) of 3.4 nM of the mAb208/HER3 complex, as described before (Fig. 2A, table 1). The dominant spot in Fig. 2B described the interaction between HER3 and mAb208, as this spot populated the same IM coordinates than in Fig. 2A, where no HRG1β was present. The spots were different in shape, indicating a higher conformational flexibility of HER3 in absence of HRG1β. The weaker spot contributed with 18% and could be ascribed to the HRG1β kinetics (Fig. 2B). It showed a *k*_a_ of 1.0E+06 M^-1^s^-1^ and a surprisingly low affinity of only *K*_D_ 110 nM (table 2), caused by its fast complex dissociation of *k*_d_ 1.1E-01 s^-1^.

To generate more detailed information about the interaction kinetics of HRG1β with HER3, SPR analyses was performed. HER3 was captured by mAb208 and HRG1β was injected subsequently (Fig. 1C). MAb208 was capture on the sensor chip surface with 256 RU and was saturated with 211 RU of HER3, followed by HRG1β injection (table 1). The Molar Ratio was calculated with MR 0.6 and indicated that every second HER3 formed a complex with HRG1β. BL_early_ was measured with 79 RU and BL_late_ with 22 RU, resulting in a signal drop of 57 RU. The first and fast dissociation phase of the SPR sensorgram migrated into a second phase of slower dissociation ending at SL 10 RU. The Langmuir fit calculated a *k*_a_ of 1.2E+08 M^-1^s^-1^ and a *k*_d_ of 4.0E-02 s^-1^, resulting in an apparent affinity of *K*_D_ 0.3 nM. A trained Biacore user as well as the integrated control software would reject the applied Langmuir fit (Fig. 1C). The Langmuir fit ignored the biphasic dissociation for the calculation of kinetic parameters and fits to the signal drop directly after the HRG1β injection, resulting in a significant failure of Chi^2^ 4.6 RU^2^ (table 1) with regards to the highest signal amplitude at R_max_ of 43 RU. Additionally, the kinetic constant *k*_a_ is exceeding the limits that can be reliably measured by the instrument. The complex character of the HER3/HRG1β interaction is proven by a control experiment (S1C Fig.). The affinity calculated by the applied two-state reaction fit was *K*_D_(2sr) 1.6 nM. The Chi^2^(2sr) value of the two-state fit was decreased by 3.6 RU^2^ to a Chi^2^(2sr) of 1.0 RU^2^ in comparison to the Langmuir fit Chi^2^ (4.6 RU^2^) (table 1).

Here, the multi-parameter IM fitting model resolved two rate contributions (Fig. 2C). The dominant spot indicated that 63% of the complex formation was characterized by an affinity of *K*_D_ 6.1 nM consisting of a *k*_a_ of 1.5E+07 M^-1^s^-1^ and a *k*_d_ of 8.9E‑02 s^-1^ (table 2). It populated the same log *k*_d_ coordinates than the weaker spot of the former assay setup (Fig. 2B), supporting the assumption that it was HRG1β-mediated. The weaker spot contributed 16% with the affinity *K*_D_ of 0.9 nM consisting of a *k*_a_ of 1.5E+06 M^-1^s^-1^ and a *k*_d_ of 1.2E-03 s^-1^ (Fig. 2C, table 2). It does not align with the dominant spot of Fig. 2B and can therefore not be ascribed to be the interaction of HER3 with mAb208. Consequently, the weaker spot could be an intramolecular avidity effect of HRG1β with HER3. The dissociation rate of HRG1β was stabilized 74-fold, increasing the affinity 7-fold from *K*_D_ 6.1 nM (dominant spot, Fig. 2C) to *K*_D_ 0.9 nM (weaker spot, Fig. 2C). IM showed, that the affinity-driven binding step dominated (63%) over the avidity-driven binding step (16%) by 3-fold.

In order to assess the affinity and avidity distribution of HRG1β in its binding mode to HER3, we performed SPR analyses in absence of mAb208. Therefore, HER3-Avi-biotin was directly presented via streptavidin, to ensure its optimal presentation with enhanced degrees of rotational freedom (Fig. 1D). 179 RU of HER3-Avi-biotin were captured and HRG1β was injected with a signal amplitude of 58 RU (BL_early_). The signal dropped by 16 RU, resulting in a BL_late_ of 42 RU. The dissociation proceeded multi-phasic to SL 30 RU at the end of the data monitoring. The Molar Ratio of 0.8 revealed functional 1:1 binding stoichiometry. The sensorgram shape and curvature completely differed from the former assay, providing support that mAb208 influenced the HER3/HRG1β complex formation. Visual inspection of the sensorgram suggested that it was too complex and therefore uninterpretable by a Langmuir fit, mainly because of the multi-phasic dissociation rate (Fig. 1D).

To avoid using a hard-to-interpret two-state reaction model, SPR experiments should be designed in a way, that a simple 1:1 fit can be applied [21]. However, experiments C and D were intentionally designed with the bivalent HRG1β molecule in solution, to mimic the *in vivo* situation. We applied a Langmuir fit regardlessly, to illustrate the complex nature of the sensorgrams. A trained Biacore user would have rejected the Langmuir model in assay D, due to the complex nature of the interaction. However, the integrated control software accepted the Langmuir fit, interpreting the signal drop as a bulk effect. Therefore, an untrained Biacore user would probably be satisfied by the quality of the fit of assay D. However, as the control experiment (S1D Fig.) shows, the HER3/HRG1β interaction is of higher complexity and a complex interaction model can be legally applied. The Langmuir fit calculated rate compositions of *k*_a_ 1.2E+07 M^-1^s^-1^ and *k*_d_ 4.6E-03 s^-1^, resulting in an affinity of *K*_D_ 0.4 nM. In comparison, the affinity of *K*_D_(2sr) 1.0 nM calculated by the two-state reaction model was 2.6-times weaker. The Chi^2^ value enhanced 3-fold from Chi^2^ 1.5 RU^2^ to Chi^2^(2sr) 0.5 RU^2^ at a highest signal amplitude of R_max_ of 56 RU.

Due to its multi-parameter fitting algorithm, the quality of the applied IM fit (Fig. 2D) was superior to the applied Langmuir fit and to the applied two-state reaction model (Fig. 1D), resulting in an increased data resolution. IM resolved three kinetic components (Fig. 2D). The dominant spot contributed with 54% and rate compositions of *k*_a_ 1.4E+06 M^-1^s^-1^ and of *k*_d_ 1.4E-03 s^-1^, leading to an affinity of *K*_D_ 1.2 nM (table 2), which was comparable to the affinity of *K*_D_(2sr) 1.0 nM. The weaker spot contributed with 27% and rate compositions of *k*_a_ 4.2E+06 M^‑1^s^-1^ and of *k*_d_ 2.5E-02 s^-1^, producing an affinity of *K*_D_ 7 nM. The weakest spot contributed with 12% and rate compositions of *k*_a_ 6.0E+04 M^-1^s^-1^ and of *k*_d_ 2.7E-04 s^-1^, leading to an affinity of *K*_D_ 4.9 nM (table 2). The comparison with the former IM (Fig. 2C) revealed, that mAb208 interfered with the avidity formation of HRG1β to HER3. In absence of mAb208, HRG1β developed its full ability to generate single digit nanomolar HER3 avidity of *K*_D_ 1.2 nM (Fig. 2D, table 2).

## Discussion

Here, we provide evidence that the activating conformational change of HER3 is ligand-induced. The finding is based on SPR data analyzed by Interaction Map and suggests a complex, avidity-driven molecular binding mode of HRG1β to HER3. Detailed interpretation of the complex kinetic interaction between HER3 and HRG1β was made possible due to the new multi-parameter fitting algorithm incorporated in Interaction Map.

When IM is applied to time-resolved interaction data one or more peaks appear in the Interaction Map. Well-defined peaks represent interaction like processes. When combining one Interaction Map with other data, such as a second IM performed under different conditions or completely other data, the nature of a peak can sometimes be deciphered. Björkelund et al [16] could link different defined peaks of EGF binding to monomeric EGFR and dimeric EGFR, and Altschuh et al [15] showed how data from a man-made heterogeneous SPR surface could be separated into the two underlying interaction components. In this paper, a similar reasoning is applied to link well-defined peaks to their molecular origin. Comparisons of Interaction Maps from similar measurements are required to form that link. The fact that peaks are representing interaction like processes means that it cannot be taken for granted that there is a monovalent Langmuir interaction taking place. Interaction Map has resolved different, non-langmuir complex interactions previously [16] and was essential to resolve the mechanism of the HER3/HRG1β interaction in this work.

We show, that the binding stoichiometry of the HRG1β/HER3 interaction in assay C was 1:2 (MR 0.5), meaning that every second HER3 interacted with one HRG1β. There are several possible explanations, like steric limitations on the sensor surface or a reduced analyte active concentration. Since the HRG1β/HER3 interaction in assay D shows a 1:1 stoichiometry, steric limitations due to the influence of mAb208 are most likely. Our data confirm that ambiguous HER3 conformations are present in equilibrium. A smaller fraction of HER3 molecules show increased mAb208 epitope accessibility and thus a faster association. Accordingly, Dawson et al. described that the main fraction of unliganded soluble HER3-ECD adopts the tethered conformation and only a small fraction is extended [22]. Once having formed a homogenous mAb208 interface, all bound receptor molecules show the same rate limiting dissociation step. When HER3 is pre-incubated with HRG1β, the interaction of the HER3/HRG1β complex with mAb208 becomes more homogenous indicating that binding of HRG1β to HER3 stabilizes one of the possible conformations. This is in accordance with literature, where the stabilization of an open, extended HER3 conformation by HRG1β binding is described [4, 6, 7].

Our data indicates that the HRG1β/HER3 complex formation is characterized by an avidity-driven molecular binding mode of HRG1β to HER3. HRG1β sequentially interacts with the HER3 binding sites. The affinity of the initial ligand:receptor contact is described by *K*_D_ 6.1 nM and switches into *K*_D_ 0.9 nM avidity for a sequential interface formation (Fig. 2C). Singer et al. conducted a HRG1β kinetic SPR evaluation with immobilized HRG1β and reported a *K*_D_ 2.3 nM avidity of HRG1β to HER3 and a 30-fold lower affinity (*K*_D_ 68 nM) for a truncated HER3, which is comprised of domains I and II and therefore lacks the second HRG1β binding site [23]. Therefore, the HRG1β/HER3 complex formation is not a simple key-into-lock docking mechanism, but at least a dynamic two state or even more probable a multi-state binding event, where the HRG1β binding valences sequentially contribute to an equilibrium formation by a complex cooperative avidity formation [7]. How such a dynamic event might take place still remains to be elucidated by additional visualizing experiments and methods.

We also provide evidence that the activating conformational change of HER3 is ligand-induced. By ranking the three association rate velocities in an assay setup which provides optimal HER3 presentation with enhanced degrees of rotational freedom, a sequential pattern of the HRG1β/HER3 docking can be ascribed. The three steps are also accompanied by an increase in the apparent complex stabilities for each of the states. Very probable and in accordance with literature HRG1β is first docking by a single valence to domain I of the tethered HER3 [7]. In the second state, HRG1β docks with a second binding site to domain III and translates into its dominating equilibrium avidity [7]. The third and slowest component could be interpreted as a final closure step, which further increases the stability of the HER3/HRG1β complex. In comparison, the homologous receptor family member HER4 [24] undergoes tremendous conformational changes of HER4 upon HRG1β binding, which Du and colleagues simulated by a one microsecond molecular dynamics simulation [7]. Du et al. claim that the overall HRG1β-induced conformational transition of the HER4 receptor consists of three stages. Therefore, HER3 might also adhere to a three-stage process upon HRG1β binding. In more detail, Du et al. claim that HRG1β (constructed by homology modeling based on the NMR coordinates of neuregulin1α (NRG1α); PDB code: 1HAF) binds initially to HER4 domain I (PDB code: 2AHX) [25, 26] and moves to domain III, due to electrostatic interactions, accompanied by domain II bending [7]. Subsequently, the domain II/IV tether is loosened and completely dissociates in the last step, whereby domains II and IV separate from each other quickly [7]. Our experimental data also showed three kinetic components in the homologous HER3/HRG1β interaction. Therefore, our data are an experimental support of what Du et al. predicted by their computational simulations.

From another angle, this work illustrates the immense power of time-resolved interaction analyses. With access to high-quality protein preparations, advanced real-time binding sensors and state-of-the-art analysis methods, complex molecular arrangements can be elucidated using only a few experimental data sets. This is in sharp contrast to the majority of the current biosensor publications where only about 20% show truly meaningful data sets in overlay with an appropriate fitting model [19]. Complex interaction data increases the level of difficulty even further and often face the scientist with insurmountable problems of how to improve the assay or how to evaluate the data in order to avoid misinterpretation of artefacts when selecting drug molecules and diagnostic reagents. We show that there is room for great improvement in this aspect.

In the recent decade high-quality proteins and high-performance real-time binding sensors have become increasingly accessible. The development of data analysis methods and the sources for adequate training has however not been equally developed. In our laboratory, Interaction Map has become a cornerstone in the scientist’s capability to develop well characterized binding molecules, especially in cases where data is complex. In this particular case, we used IM to resolve the rate limiting kinetic contributions from complex Biacore sensorgrams. Applying a two-state reaction model to complex SPR data requires proof of the complex nature of the investigated interaction. Here, the applied two-state reaction fits resulted in affinities similar to those calculated by IM, showing the comparability of both analysis tools. Adding to the value of IM, the generated topographic maps made the complex character of the interactions visible and interpretable even for untrained Biacore users in a manner that regular analysis software is incapable of. We could resolve details from the complex, avidity-driven binding mode of Heregulin 1 beta (HRG1β) with the HER3 receptor extracellular domain (HER3). The presented SPR-derived insights support the idea of a complex, avidity-driven molecular binding mode of HRG1β to HER3, in a way Du et al. proposed for the HER4/HRG1β system. We are therefore in favor of a model where the activating conformational change of HER3 is ligand-induced.

## Conclusions

We provide evidence that the activating conformational change of HER3 is ligand-induced. The finding is based on SPR binding data analyzed by Interaction Map and suggest a complex, avidity-driven molecular binding mode of HRG1β to HER3.

## Supporting Information

S1 Fig Confirm or disconfirm the complex character of interactions, by using a complex interaction analysis. Using a Biacore 4000 device (GE Healthcare) the monophasic or complex character of different interactions was identified, by varying the association phase time [20]. Three replicates of all experiments were conducted and the sensorgrams show an overlay of three replicates (black) and, where applicable, the corresponding 1:1 Langmuir fit (red) of the dissociation phase. The assay setup is shown in the left panel. The middle panel shows the overlay sensorgrams and in the right panel the data were normalized by setting the response at the start of the dissociation phase to 1 [20]. **(A)** The overlay of the dissociation phase indicates the HER3/mAb208 interaction is independent of the contact time. The monophasic interaction can be fitted by a 1:1 Langmuir model. **(B)** The curves are congruent, but complex in their dissociation behavior. They are not fittable by a Langmuir model. (**C and D**) Since the dissociation phases were incongruent at different injection times, a complex character of the HRG1β/HER3 interaction was ascertained. The data could not be fitted by a 1:1 Langmuir model as well.
(TIF)
