# Supplementary figures and images for: Deciphering the Stepwise Binding Mode of HRG1β to HER3 by Surface Plasmon Resonance and Interaction Map
Source: PLoS One. 2015 Feb 6;10(2):e0116870. doi: 10.1371/journal.pone.0116870 (PMC4319926; doi:10.1371/journal.pone.0116870)

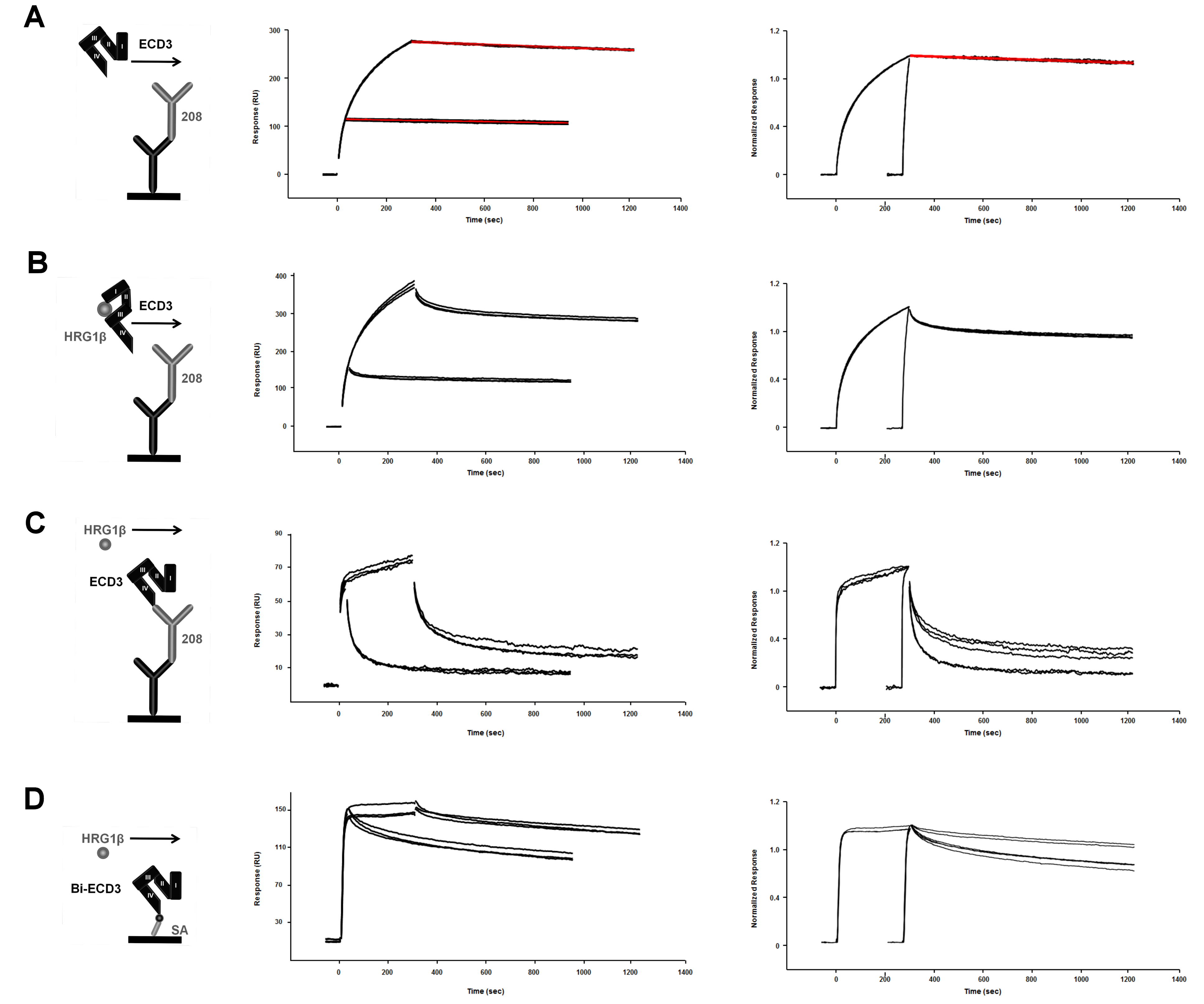

Supplement: S1 Fig — Using a Biacore 4000 device (GE Healthcare) the monophasic or complex character of different interactions was identified, by varying the association phase time [20]. Three replicates of all experiments were conducted and the sensorgrams show an overlay of three replicates (black) and, where applicable, the corresponding 1:1 Langmuir fit (red) of the dissociation phase. The assay setup is shown in the left panel. The middle panel shows the overlay sensorgrams and in the right panel the data were normalized by setting the response at the start of the dissociation phase to 1 [20]. (A) The overlay of the dissociation phase indicates the HER3/mAb208 interaction is independent of the contact time. The monophasic interaction can be fitted by a 1:1 Langmuir model. (B) The curves are congruent, but complex in their dissociation behavior. They are not fittable by a Langmuir model. (C and D) Since the dissociation phases were incongruent at different injection times, a complex character of the HRG1β/HER3 interaction was ascertained. The data could not be fitted by a 1:1 Langmuir model as well. (TIF) [file pone.0116870.s001.tif]
